# Effects of Abiotic Elicitors on Expression and Accumulation of Three Candidate Benzophenanthridine Alkaloids in Cultured Greater Celandine Cells

**DOI:** 10.3390/molecules26051395

**Published:** 2021-03-05

**Authors:** Seyed Mohammad Hashemi, Mohammad Reza Naghavi, Esmaeil Bakhshandeh, Mehdi Ghorbani, Chanditha Priyanatha, Peiman Zandi

**Affiliations:** 1 Department of Plant Agriculture, Ontario Agriculture College, University of Guelph, 50 Stone Road East, Guelph, ON N1G 2W1, Canada; cpriyana@uoguelph.ca; 2 Department of Agronomy and Plant Breeding, College of Agriculture and Natural Resources, University of Tehran, Karaj 31587-77871, Iran; mghorbani_90@ut.ac.ir; 3 Genetics and Agricultural Biotechnology Institute of Tabarestan, Sari Agricultural Sciences and Natural Resources University, Sari 4818166996, Iran; e.bakhshandeh@sanru.ac.ir; 4 Julius Kühn-Institut, Federal Research Centre for Cultivated Plants, Institute for Crop and Soil Sciences, Bundesallee 69, 38116 Braunschweig, Germany; z_rice_b@yahoo.com; 5 International Faculty of Applied Technology, Yibin Unversity, Yibin 644000, China

**Keywords:** benzophenanthridine alkaloids, *Chelidonium majus* L., elicitation, secondary metabolites, transcription regulation

## Abstract

Efforts to develop the necessary biotechnologies in Greater Celandine (*Chelidonium majus* L.), a leading plant resource for the development of plant-derived medicines, have been hampered by the lack of knowledge about transcriptome and metabolome regulations of its medicinal components. Therefore, this study aimed to examine the effect of abiotic elicitors, methyl jasmonate (MJ) and salicylic acid (SA), at different time courses (12, 24, 48, and 72 h), on expression and metabolome of key benzophenanthridine alkaloids (BPAs) in an optimized in vitro culture. Gene expression analysis indicated the upregulation of *CFS* (cheilanthifoline synthase) to 2.62, 4.85, and 7.28 times higher than the control at 12, 24, and 48 h respectively, under MJ elicitation. Besides, MJ upregulated the expression of *TNMT* (tetrahydroprotoberberine *N*-methyltransferase) to 2.79, 4.75, and 7.21 times at 12, 24, and 48 h respectively, compared to the control. Investigation of BPAs revealed a significant enhancement in the chelidonine content (9.86 µg/mg) after 72 h of MJ elicitation. Additionally, sanguinarine content increased to its highest level (3.42 µg/mg) after 24 h of MJ elicitation; however, no significant enhancement was detected in its content in shorter elicitation time courses. Generally, higher gene expression and BPAs’ level was observed through longer elicitation courses (48 and 72 h). Our findings take part in improving the understanding of transcription and metabolic regulation of BPAs in cultured Greater Celandine cells.

## 1. Introduction

Greater Celandine (*Chelidonium majus* L.) is a biennial/perennial plant native to Europe and North Asia [1]. It is a promising medicinal plant in terms of traditional and modern pharmacology, which covers a range of valuable phytochemicals, most notably anti-cancer chelidonine [2], anti-inflammatory berberine [3], and anti-bacteria sanguinarine [4]. This plant is well-known for its lead in the industry of anti-cancer plant-derived medicines [5,6]. Ukrain^TM^, derived from secondary metabolites (SMs) of Greater Celandine, is extensively accepted as a pharmaceutically active and clinically effective anti-cancer medicine [6]. To produce SMs for medicinal purposes, most plant-based pharmaceutical industries are propagating plants through traditional manners. They are planting them on the farm and extracting medicinal components directly from intact plants, which completely depends on the growing location and harvesting season [7].

Plant cell culture, on the other hand, is introduced as a potential alternative to up-scale production of SMs in a shorter time and less space than traditional manners [7]. Moreover, a higher level of SMs production of different medicinal plants through cell culture has already been reported [8,9], although the production level of SMs through cell culture is still considerably lower than the intact plant in some cases. Particular substances called “elicitors” are applied as stress agents to boost the SM production in cultured plant cells [10]. Application of these substances to stimulate the production of SMs in plant cells is called “elicitation” and it initiates by an attack on the plant cell, then triggers a cascade of signal transduction pathways and finally leads to enhance expression of related genes and improve SMs accumulation [11,12]. 

**Figure 1:**
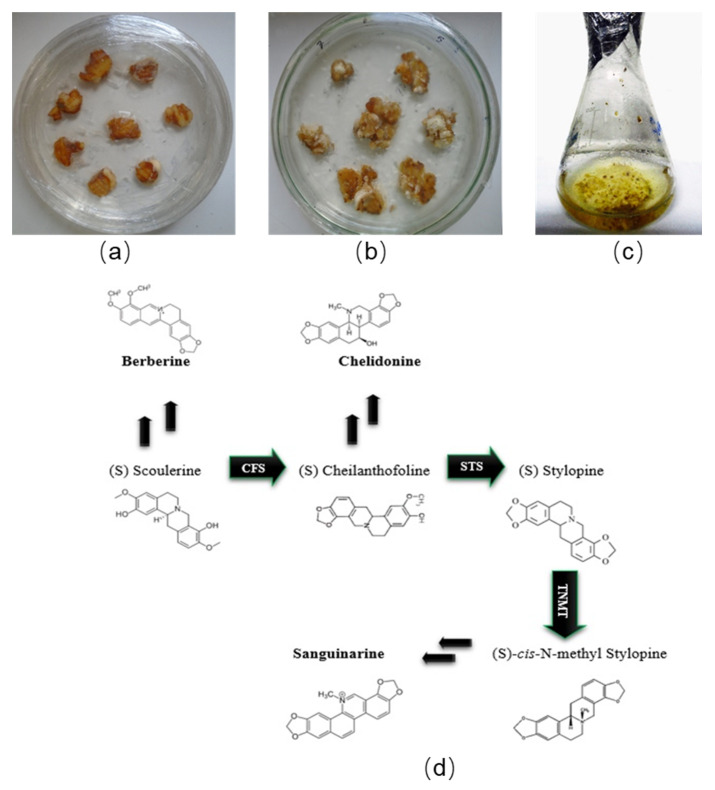
Callus and cell suspension culture of Greater Celandine. (**a**) The initiation phase of calli in leaf explants on Murashige and Skoog (MS) medium supplemented with 1 mg L^−1^ of indole-3-acetic acid (IAA) and 0.2 mg L^−1^ of 6-benzylaminopurine (BAP). (**b**) Callus culture on MS medium supplemented with 1 mg L^−1^ IAA and 0.2 mg L^−1^ BAP. (**c**) Cell suspension culture of Greater Celandine. (**d**) The metabolic pathway of benzophenanthridine alkaloids that illustrates the role ofCFS (cheilanthifoline synthase), STS (stylopine synthase), and TNMT (tetrahydroprotoberberine *N*-methyltransferase) enzymes [19,20].

Methyl jasmonate (MJ) and salicylic acid (SA) are two well-known elicitors and pivotal defense signaling molecules that can trigger a stress-response cascade, resulting in improvement of the SMs accumulation [13]. Almost all SM biosynthetic pathways, including benzophenanthridine alkaloids (BPAs), are induced by the application of jasmonates (JA) or endogenous enhancement in JA [14,15]. Accumulation of BPAs has also been improved in cell suspension cultures of *Rauvolfia canescens* and *Eschscholtzia californica*, well-known medicinal plants, after elicitation with JA [15]. These elicitors, MJ and SA, also play a crucial role in stimulating the expression of SMs-related genes [16,17]. There are seven reactions in the biosynthetic pathway of BPAs [18] and three of them: *CFS* (cheilanthifoline synthase), *STS* (stylopine synthase), and *TNMT* (tetrahydroprotoberberine *N*-methyltransferase), are key in the regulation of BPAs [19,20] (Figure 1).

Previous studies from our laboratory demonstrated that MJ and SA play central roles as abiotic stimulators in improving the accumulation and gene expression of SMs such as morphine, codeine, artemisinin, etc. However, the role of these stimulators in the production and gene expression of BPAs using cell suspension culture of Greater Celandine remains unclear. Consequently, the main objective of this study is to investigate the effect of MJ and SA on the biosynthesis of BPAs and the expression of their related genes at different time courses. Furthermore, the present study also aimed to analyze the role of different tissue sources, media type, auxins, and cytokinins in callus induction of Greater Celandine since callus culture optimization is a prerequisite for the propagation process of cell culture.

## 2. Results

### 2.1. Propagation Optimization

**Figure 2:**
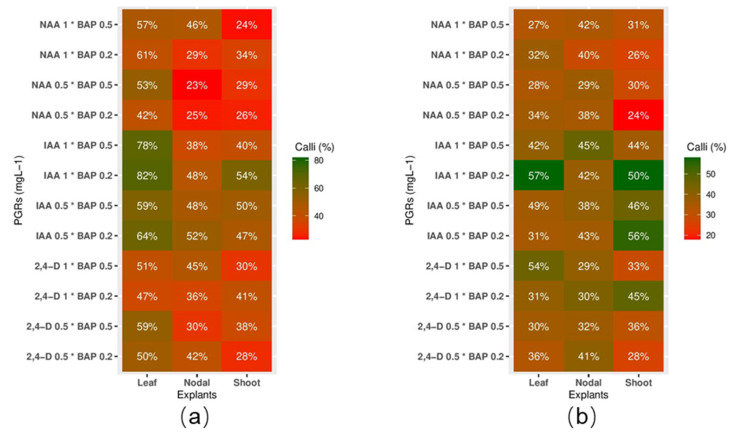
Callus induction frequency of Greater Celandine. (**a**) Callus induction rate using different plant growth regulators PGRs and explants on MS medium. (**b**) Callus induction rate using different PGRs and explants on B5 media. Each cell in the heatmap represents the mean value of six replicates. * represents the combination of PGRs.

Two types of media, MS (Murashige and Skoog) and B5 (Gamborg B5), three sources of explants (leaf, shoot, and nodal), and four types of plant growth regulators (PGRs), IAA (indole-3-acetic acid), NAA (1-naphthaleneacetic acid), 2,4-D (2,4-dichlorophenoxyacetic acid), and BAP (6-benzylaminopurine), were tested to uncover the most efficient platform for callus induction of Greater Celandine. Figure 2 illustrates the callus induction frequency under different treatments. It was revealed that MS medium is significantly more favorable than B5 medium to produce and propagate a callus. Compared to nodal and shoot tissue, leaf explants, with 59.13% of callus induction on the MS media, were revealed to be the more favorable tissue source to initiate callus formation (Figure 2a). A closer look at the data indicates that the combination of 1 mg L^−1^ IAA and 0.2 mg L^−1^ BAP on the MS medium for leaf, nodal, and shoot explants with 82%, 48%, and 54% respectively, was more effective than other PGR combinations (Figure 2a). The B5 basal medium supplemented with 0.5 mg L^−1^ IAA and 0.2 mg L^−1^ BAP was found to be the most promising platform for shoot explants, with 56% of callus induction frequency (Figure 2b). For nodal explants, moreover, the highest percentage of callus induction (52%) was achieved on MS medium supplemented with 0.5 mg L^−1^ IAA and 0.2 mg L^−1^ BAP. The highest number of callus initiation was obtained on MS medium supplemented with 1 mg L^−1^ IAA and 0.2 mg L^−1^ BAP using leaf as explant, whereas the lowest percentage was detected on MS medium supplemented with 0.5 mg L^−1^ NNA and 0.5 mg L^−1^ BAP using nodal as explant.

### 2.2. Cell Growth

**Figure 3:**
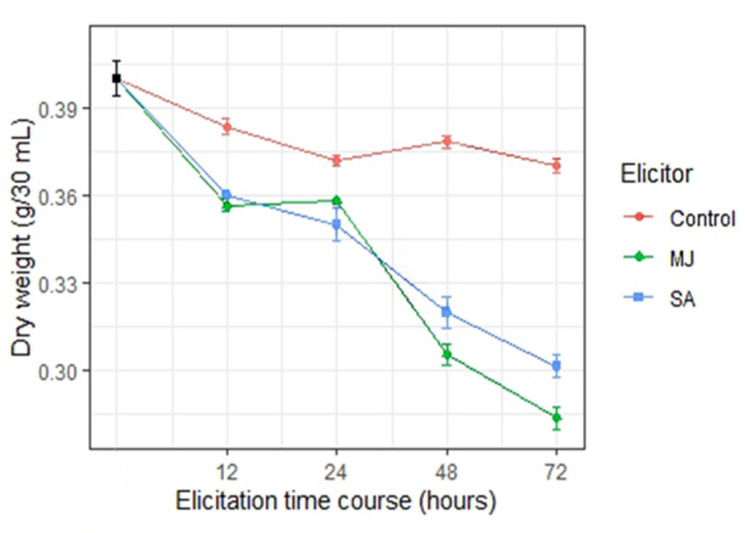
Effects of methyl jasmonate (MJ) and salicylic acid (SA) on cell growth of Greater Celandine at different time courses (12, 24, 48, and 72 h). Dry weight of cells after 14 days of the culture cycle (stationary growth phase) is represented by the black point. Non-elicited cells were used as control. Each data represents the mean value of six replications and error bars represent mean ± standard deviation (SD).

Dry cell weight decreased remarkably as the elicitation time courses of elicitors increased (Figure 3). Significant effects were observed during different time courses in dry cell weight via elicitation of both elicitors in comparison with the controls. Both MJ and SA significantly decreased the dry cell weight at all elicitation time courses. Nonetheless, longer time elicitations, 48 and 72 h, indicated more shrinkage in dry cell weight than shorter times, 12 and 24 h. SA elicitation decreased dry cell weight from 40.13 to 32.23 and 30.07 g/30 mL at 48 and 72 h, respectively. In contrast to SA, MJ elicitation with 30.21 and 28.44 g/30 mL at 48 and 72 h respectively, presented a higher influence in diminishing dry cell weight.

### 2.3. Accumulation of BPAs

**Figure 4:**
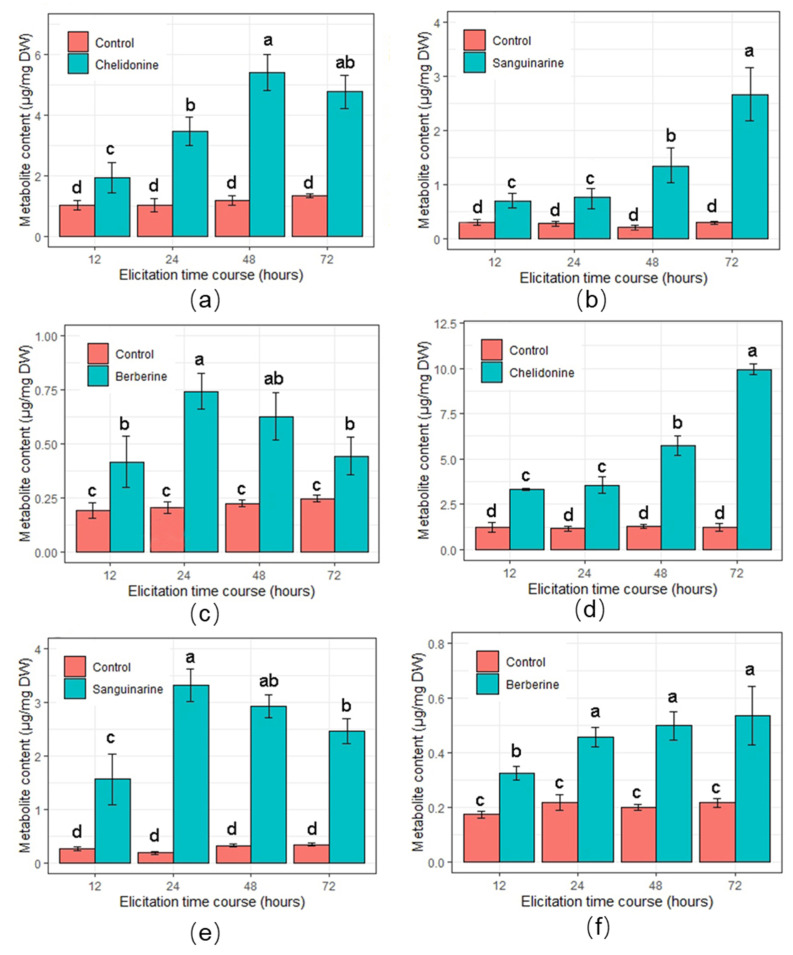
Effects of abiotic elicitors on the accumulation BPAs in cultured Greater Celandine cells. (**a**) chelidonine, (**b**) sanguinarine, and (**c**) berberine after elicitation by SA at 12, 24, 48, and 72 h. (**d**) Chelidonine, (**e**) sanguinarine, and (**f**) berberine after elicitation by MJ at 12, 24, 48, and 72 h. Non-elicited cells were used as control. Each column represents the mean value with the SD bar from three biological and three technical replicates. The significant difference is calculated by LSD (least significant difference) statistical analysis at the level of *p* ≤ 0.05.

The BPAs content of the treated and control cultures was measured after different times of elicitation. High-performance liquid chromatography (HPLC) uncovered significant improvements in the biosynthesis of BPAs under MJ and SA elicitations compared to control at almost all different time courses (Figure 4). The accumulation of chelidonine improved by 2.52-fold (5.61 µg/mg) after 48 h of SA elicitation compared with the control (Figure 4a). A significant improvement was observed in the content of sanguinarine (2.69 µg/mg) after 72 h of SA elicitation (Figure 4b). The accumulation of berberine after 24 h of SA elicitation reached the maximum point of 4.89-fold (0.75 µg/mg) in comparison with the control (Figure 4c). MJ-treated cells produced a higher level of chelidonine and sanguinarine compared to SA-treated ones (Figure 4d,e). The amount of chelidonine after 72 h of MJ elicitation was considerably increased by 9.63-fold (9.86 µg/mg) to its highest point (Figure 4d). After 24 h of MJ elicitation, the highest level of accumulation was observed in sanguinarine content by 13.22-fold (3.42 µg/mg), in comparison with control (Figure 4e).

### 2.4. Expression of BAPs Biosynthetic Genes

**Figure 5:**
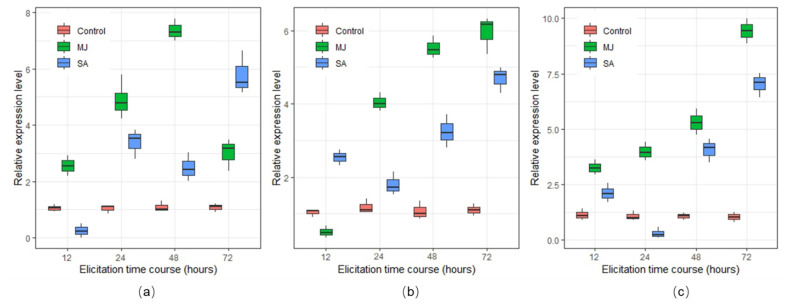
Effects of MJ and SA elicitation, at different time courses, on the expression of nominated benzophenanthridine alkaloids genes in cultured Greater Celandine cells. (**a**) *TNMT,* (**b**) *STS*, and (**c**) *CFS*. Non-elicited cells were used as control. The concentration of MJ and SA was 100 µM. *EF1-ɑ* was used as an internal control to normalize variation in the amount of cDNA template. Each data point represents the mean value of three biological and three technical replications.

To understand the mechanism of the BPAs production in cell suspension culture of Greater Celandine, expression level of key genes (*CFS*, *STS*, and *TNMT*) which are involved in BPAs biosynthesis was studied using quantitative real time polymerase chain reaction (qRT-PCR). Quantitative transcriptome analysis demonstrated that MJ and SA, at all the time courses, stimulated the transcription level of the genes in BPAs biosynthetic pathway. A notable association was detected between the expression level of candidate genes and the accumulation of BPAs. MJ has been noticeably effective on the upregulation of *TNMT* at 12, 24, and 48 h elicitation and enhanced the expression level of this gene by 2.79, 4.75, and 7.21 times respectively, in comparison with the control (Figure 5a). However, in comparison with 24 and 48 h, a significant downregulation was observed in the expression level of *TNMT* at 72 h MJ elicitation. In contrast with MJ, a significant downregulation at 12 h and upregulation at 72 h was shown in the expression level of *TNMT* after SA elicitation. Improvement in the expression of *STS* was observed by MJ elicitation at any time course excluding 12 h and it reached the highest point at 6.17 times higher than the control after 72 h of elicitation (Figure 5b). In SA elicitation, an upregulation trend was observed in the expression of *STS* by increasing elicitation time courses, except for 12 h. SA was also significantly effective on the downregulation of *CFS* at 24 h and decreased its expression level by 1.06 times compared to the control. However, MJ elicitation upregulated the transcription level of *CFS* by 2.62, 4.85, and 7.28 times higher than control at 12, 24, and 48 h, respectively (Figure 5c). In general, longer time elicitations through MJ and SA could improve the expression level of key genes in the BPAs pathway.

## 3. Discussion

In the present study, the discussion centers on improving the biosynthesis of plant-derived pharmaceutically active components through biotechnologies. For quite a while, extraordinary efforts have been devoted to the investigation of the biosynthesis of plant-derived bioactive components [21,22,23]. BPAs are of leading deliberation in traditional and modern pharmacology due to demonstrating pharmaceutically active features [19]. The high rate of cell growth and the good reproducibility of cells in suspension culture make it a suitable platform for the up-scale production of valuable SMs. The elicitation of cells in suspension culture is a more advanced strategy to improve the production of these highly valuable SMs [24]. Having an optimal callus growth platform would thus be a prerequisite to establishing cell suspension culture and further assessment of transcriptome and metabolome SMs [7].

Here, an appropriate cell growth platform was established to assess the expression and accumulation of BPAs in Greater Celandine. Two different media, MS and B5, were used to test callus initiation. MS and B5 media, due to their nutrition components, were previously documented as favorable media for callus induction of various medicinal plants, including *Barringtonia racemosa* L. and *Allium chinensis* G. Don. Our results disclosed that the MS medium is the most favorable for callus induction of Greater Celandine. Because of its higher concentration of nitrate, MS medium can improve cell growth and have a direct linkage with callus proliferation [25]. The type of explant is considered a vital factor in callus initiation as well [26]. Previous studies on in vitro proliferation of medicinal plants have described leaf explants as a suitable source for callus initiation and it is mainly due to the low level of SMs in leaves [26,27,28]. Leaf explants demonstrated the highest percentage of callus induction here as well. Eventually, among the PGRs, the combination of 1 mg L^−1^ IAA and 0.2 mg L^−1^ BAP were considerably more influential than others. It has already been reported that IAA, as a natural auxin, can induce higher callus formation than synthetic auxins [29,30].

To conduct elicitation, cell suspension cultures were developed from calli. Cell suspension culture has proven to be a superior approach for improving SMs by elicitation in comparison with callus culture [24,31]. Two commonly used abiotic elicitors, MJ and SA, were found to be effective in triggering stress in cultured Greater Celandine cells. MJ and SA are involved in plant defense systems against stresses [32,33]. SA elicitation of *Panax ginseng* cells resulted in a reduction of cell weight by approximately 25% after nine days [34]. Furthermore, 9.25-fold L-Dopa content has been achieved in *Hybanthus enneaspermus* cultured cells with 150 µM SA elicitor [35]. MJ has a similar effect as osmotic stress on plant cells [36]. Cell growth rate indicated that increasing the elicitation time course increased osmotic stress and resulted in cell degradation and decrement in cell growth accordingly. Another study on *Taxus* cells showed decreasing plant cell weight through elicitation as well [37]. Our findings indicated a significant reduction of cell weight through longer-term MJ-elicitation, suggesting that MJ treatment increases osmotic stress more than treatment with SA in longer elicitation times.

During the elicitation process, biosynthesis of SMs is controlled by various factors, such as elicitor specificity, elicitation exposure time, age of culture, etc. [38,39]. The application of exogenous MJ and SA have been well-recognized to stimulate the accumulation of SMs in plant cells. However, the effect of exogenous MJ and SA on the accumulation of BPAs in Greater Celandine cells has not been studied yet. Therefore, in the present study, both MJ and SA were identified as considerably effective abiotic elicitors in the biosynthesis of BPAs in Greater Celandine cells. MJ plays a fundamental role in inducing defense mechanisms that are biologically effective on the regulation of the signaling network leading to the biosynthesis of SMs [40,41]. Consequently, it was possible to observe MJ to serve as an effective elicitor in Greater Celandine cells by triggering the accumulation of BPAs as well as diminishing cell growth rate in comparison to the control.

A positive tendency of BPAs biosynthesis was also observed in SA-elicited cells but not in substantial quantities in comparison to MJ-elicited cells. Similar results have been observed in the cell suspension culture of *Hypericum perforatum* and *Thevetia peruviana*, and MJ improved biosynthesis of phenolic components when compared with SA elicitation [31,42]. Furthermore, in the elicitation approach, the duration of the elicitor exposure as well as the type of elicitor are two key factors that stimulate the biosynthesis of SMs [24]. Under both elicitor treatments, the greater production level of BPAs and lower cell growth rate were observed at longer time courses, indicating that longer time elicitations were more influential. Earlier studies reported SA and MJ as effective elicitors in improving the production of SMs at longer time courses [34]. However, biosynthesis of berberine and sanguinarine diminished by SA and MJ elicitation respectively, at 48 and 72 h. It may be due to the lower activity level of *TNMT* enzyme or low expression level of *TNMT* gene at 48 and 72 h after MJ elicitation. Similar studies have documented that MJ and SA can activate and/or deactivate the expression level of associated genes with SMs in plant cells [43,44,45].

MJ and SA generally upregulated the expression of key genes *CFS*, *STS*, and *TNMT*, enhanced the accumulation of BAPs, and reduced cell growth rate. Such genes have already been well-described to play a leading role in the biosynthesis of BPAs [19]. The expression levels of *CFS* and *STS* were upregulated by longer elicitation times. However, *TNMT* was downregulated by MJ and SA at 72 and 48 h, respectively. This variation in the expression of genes was found to be associated with the content of sanguinarine. It has already been reported that the biosynthesis of sanguinarine has a strong association with the expression level of *CFS*, *STS*, and *TNMT* [20]. CFS is the upstream enzyme of STS and TNMT in the benzophenanthridine alkaloids pathway [19]. Consequently, once the expression of *CFS* was upregulated by MJ at 72 h, the content of chelidonine was enhanced, implying that the biosynthesis of chelidonine is associated with the expression level of *CFS*. Similar results were observed regarding the content of sanguinarine. Its content reduced when the expression of *TNMT* was downregulated, suggesting that this downregulation had an association with the accumulation of sanguinarine. Related findings have been well-documented that SA improves the expression of isoquinoline alkaloids and the biosynthesis of related metabolites accordingly [20]. Nevertheless, SA has not been more effective in the production of chelidonine, which is most probably due to the expression levels of *CFS*. Since the content of alkaloids increased via increasing expression of related genes, it was detected that there might be a strong linkage between the gene’s expression and the production of BPAs. Moreover, MJ and SA enhanced the content of chelidonine through the upregulation of *CFS* at all of the elicitation time courses. It has already been reported that upregulation of *CFS* can result in increasing the biosynthesis of chelidonine [46].

## 4. Materials and Methods

### 4.1. Plant Materials and Seed Germination

Seeds of Greater Celandine were obtained from the “Medicinal Herb Seed Bank (www.seedsnow.com) (accessed on 15 February 2015)”. The seeds were surface-sterilized with 70% (*v*/*v*) ethanol for 1 min and 2.5% (*v*/*v*) sodium hypochlorite for 10 min, then rinsed three times in sterile distilled water. Subsequently, the sterilized seeds were germinated on basal medium containing B5 salts and vitamins [47] with 3% sucrose (*w*/*v*) and solidified with 7% (*w*/*v*) agar. Finally, shoots, leaves, and nodals from forty-day-old plants were used as explants.

### 4.2. Callus and Cell Suspension Culture

Different explants including leaf, shoot, and nodal were placed on two different media—B5 and MS [48]—supplemented with 12 different combinations and concentrations of PGRs (IAA, BAP, NAA, and 2,4-D). In addition, each plate was supplemented with 3% sucrose (*w*/*v*), 0.5% ascorbic acid (*v*/*v*), 0.5% polyvinylpyrrolidone (*w/v*), and 7% agar (*w*/*v*). The pH of the solutions was adjusted to 5.8 and then sterilized by autoclaving at 121 °C for 20 min. PGRs were filter-sterilized via 0.22 µm cellulose acetate syringe filters and added to each base medium. To initiate calli, different explants were sub-cultured every 20 days and placed in a dark culture room at 22 ± 0.5 °C (Figure 1). Six replications were applied for each treatment.

For the development of cell suspension cultures, 1 ± 0.05 g of calli were cultivated in 100 mL Erlenmeyer flasks containing 30 mL liquid medium, the same as callus culture media without agar and the same combination and concentration of PGRs (Figure 1c). The flasks were incubated in a rotary shaker (110 rpm) at 22 ± 0.5 °C in dark conditions. Then, the cell suspension cultures were sub-cultured up to reaching homogeneity.

### 4.3. Elicitation Treatments

Elicitation treatment was carried out using MJ and SA (Sigma-Aldrich Co., Darmstadt, Germany) after 14 days (stationary growth phase) from the establishment of cell suspension culture. The stock solutions of MJ and SA were prepared in 99.9% ethanol. Subsequently, both solutions were sterilized by filtration (0.22 µm cellulose acetate syringe filters) and added to the flasks. The final concentration of elicitors after adding to flasks, containing 30 mL liquid medium and 1 ± 0.05 g of calli, was 100 μM. Afterwards, each flask was incubated at the same conditions for 12, 24, 48, and 72 h. Filter-sterilized ethanol was added to the cell suspension culture as a control.

### 4.4. Measurement of Cell Growth

The cell growth was determined by measuring the dry cell weight after the 14th day of the culture cycle, stationary growth phase. Briefly, the separation of biomass from medium (each flask) was completed by filtration. Samples were washed several times by distilled water to eliminate the remaining medium from the cells. Later, via freeze drier, the biomass was lyophilized to obtain constant weight.

### 4.5. Metabolite Extraction and Evaluation

Freeze-dried cells, 50 mg of each sample, were prepared with 1 mL of 99.9% methanol for 12 h, followed by ultrasonic extraction (WiseClean^®^) for 60 min at room temperature. The extract was centrifuged at 11,000× *g* for 8 min. The supernatant was transferred to a new tube and dried at 55 °C. The residue was dissolved with 900 µL of HCl-methanol 25% hydrolyzed at 90 °C for 30 min, and then brought to 1 mL using methanol [35].

**Figure 6:**
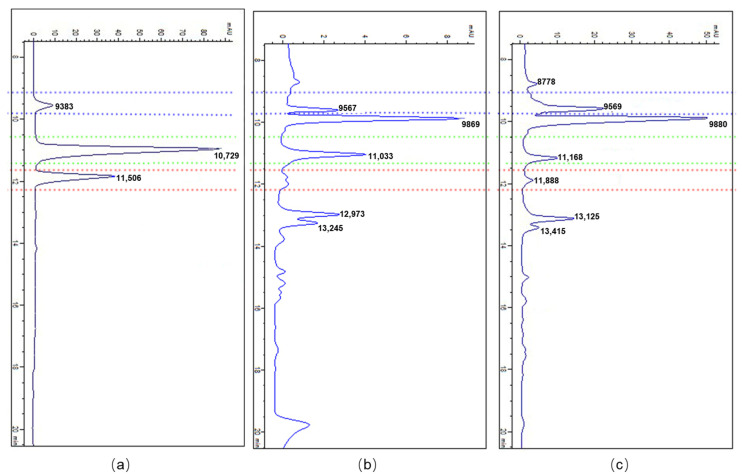
Retention time and absorption spectrum of BAPs and their external standards, as determined by high-performance liquid chromatography (HPLC). (**a**) HPLC chromatogram of the external standard of chelidonine, berberine, and sanguinarine, (**b**) HPLC chromatogram of the extract of cultured cells as a control sample, and (**c**) HPLC chromatogram of the cell extraction after 72 h of MJ elicitation. Retention time and peak shape of chelidonine, berberine, and sanguinarine are represented by blue, green, and red dotted lines, respectively.

To determine the content of BPAs, high-performance liquid chromatography (HPLC) (Knauer, PLATIN blue system, Berlin, Germany) was used. BPAs were identified through comparison of their retention time and absorption spectrum of their standard solution (Figure 6). A C18 column (Eurosphere, 250 × 4.6 mm, 5 μm) with the mobile phase of 0.02 M KH_2_PO_4_-Acetonitrile (95:5 *v*/*v*) was used as an analytical column. The flow rate was 1 mLmin^−1^ with Ultraviolet (UV) detection wavelength at 280 nm and the column temperature was 25 °C. To obtain a calibration curve, different concentration of standard solutions of BPAs (Sigma–Aldrich) were made via 99.9% methanol (Sigma–Aldrich). Ultimately, the quantification of each BPA was determined via the calibration curves of standards. Three biological and three technical replications were applied in metabolome evaluation and the mean value of replications was used to display results.

### 4.6. RNA Extraction and Transcription Analysis

Powered cells (250 mg) with various elicitation treatments at the nominated time courses (12, 24, 48, and 72 h) were collected for RNA extraction. The phenol-based method according to the manufacturer’s instruction (TRIzol^®^) followed by subsequent treatment with DNase I (Thermo Fisher Scientific, Waltham, MA, USA) was used for the extraction of total RNA from the cells. Spectrophotometry (Nano-Drop, Technologies Inc., Waltham, MA, USA) and Qubit^®^ (Thermo Fisher Scientific) were used to assess the quantity and quality of RNA. RNA integrity was evaluated from the 25S and 18S ribosomal RNA bands employing 1% non-denatured agarose gel electrophoresis. Later, 1 µg of total extracted RNA was used to produce cDNA through the oligo (dT) 18 and random hexamer primer using the first-strand cDNA synthesis kit (Thermo Fisher Scientific). The primer sequences were designed on the foundation of the corresponding genes through Primer Quest software (http://www.idtdna.com/pages/tools/primerquest) (accessed on 15 March 2016). The length of the target fragments, annealing temperature, and qRT-PCR condition are illustrated in Table 1. The reference gene was *Elongation factor 1-alpha* (*EF1*-a) (Table 1).

Amplification assay was performed in a total reaction volume of 20 μL containing 8 μL Eva-Green Master Mix (containing Eva-Green Dye, Solis BioDyn, Düsseldorf, Germany), 1.5 μL of amplified cDNA, 0.25 μM of each primer, followed by adding PCR-grade water. The qRT-PCR was completed in the Rotor-Gene Q thermocycler (Qiagen Co., Hilden, Germany) as follows: 5 min pre-denaturation at 94 °C, 1 cycle; 10 s denaturation at 94 °C, 20 s annealing temperature, 15 s collection fluorescence at 72 °C, 35 cycles. The gene expression quantification was carried out by the 2^−∆∆Ct^ method [49]. In this means, the CT (the threshold cycle above which the boost in fluorescence is exponential) values of the target and control genes are standardized to an internal standard or reference. Besides, each data point represents the average of three biological and three technical replications.

### 4.7. Statistical Analysis

The experiment was carried out based on a factorial arrangement in a completely randomized design. All statistical analyses were performed using the R agricolae package [50]. The data were analyzed by one-way analysis of variance (ANOVA) and all analytical values represent the averages of three biological and three technical replications. Visualization of data was accomplished using the R ggplot2 package [51]. The least significant difference (LSD) test at *p* < 0.05 was used to calculate the difference between averages of the samples.

## 5. Conclusions

To design strategies to improve the production of bioactive compounds, an understanding of how plant cells at the transcriptome and metabolome levels respond to abiotic elicitors is a prerequisite. One of the main obstacles for the in vitro production of BPAs is the limited knowledge of biosynthetic pathways and their controlling enzymes and genes. Here, we addressed this challenge by testing two abiotic elicitors, MJ and SA, to establish their impact on transcription and accumulation of three key BAPs in Greater Celandine. Our results revealed that the accumulation of BPAs improved by the upregulation of candidate genes, *CFS*, *STS* and *TNMT*, after MJ and SA elicitation. Our findings would be an advantage in bioengineering of the BPAs pathway and up-scale production of BPAs in Greater Celandine cell suspension culture.

## Figures and Tables

**Table 1:** Primers for target genes used for the quantitative real time polymerase chain reaction (qRT-PCR).

Gene	Accession Number	Primer Sequence *(5′*–*3′)*	Amplicon Length (bp)
*CFS*	GU325749.1	F-GAAAAGGTCTTCAAGGTGTTGC R-GAGACGGTTCGATTACTAAGTCG	150
*STS*	GU325750.1	F-TGGATCGGAAGTTGGAGAC R-GCCACATTTTGAAGACCTTTTCG	187
*TNMT*	EU882994.1	F-GCAGTCGACGAGGATGACTGG R-GTGCATTCCGTTCACAACCCAATGATC	107
*EFT-α*	XM026563191.1	F-AGATGATTCCAACCAAGCCC R-CCTTGATGACACCAACAGCA	113

## Data Availability

Not applicable.

## Sample Availability

Samples of the compounds are not available from the authors.
