# Correction: Hashemi et al. Effects of Abiotic Elicitors on Expression and Accumulation of Three Candidate Benzophenanthridine Alkaloids in Cultured Greater Celandine Cells. *Molecules* 2021, *26*, 1395

**DOI:** 10.3390/molecules27165244

**Published:** 2022-08-17

**Authors:** Seyed Mohammad Hashemi, Mohammad Reza Naghavi, Esmaeil Bakhshandeh, Mehdi Ghorbani, Chanditha Priyanatha, Peiman Zandi

**Affiliations:** 1 Department of Plant Agriculture, Ontario Agriculture College, University of Guelph, 50 Stone Road East, Guelph, ON N1G 2W1, Canada; 2 Department of Agronomy and Plant Breeding, College of Agriculture and Natural Resources, University of Tehran, Karaj 31587-77871, Iran; 3 Genetics and Agricultural Biotechnology Institute of Tabarestan, Sari Agricultural Sciences and Natural Resources University, Sari 4818166996, Iran; 4 Julius Kühn-Institut, Federal Research Centre for Cultivated Plants, Institute for Crop and Soil Sciences, Bundesallee 69, 38116 Braunschweig, Germany; 5 International Faculty of Applied Technology, Yibin Unversity, Yibin 644000, China

The authors wish to make the following correction to the paper [1]: During the final preparation of the manuscript, name and affiliation of the third author Esmaeil Bakhshandeh was unintentionally omitted, his contribution is editing/reviewing and validation in this paper. We would like to add his name and affiliation: Genetics and Agricultural Biotechnology Institute of Tabarestan, Sari Agricultural Sciences and Natural Resources University, Sari 4818166996, Iran. The correct authors and affiliations are as follows:

Seyed Mohammad Hashemi ^1,^*, Mohammad Reza Naghavi ^2,^*, Esmaeil Bakhshandeh ^3^, Mehdi Ghorbani ^2^, Chanditha Priyanatha ^1^ and Peiman Zandi ^4,5^
  ^1^  Department of Plant Agriculture, Ontario Agriculture College, University of Guelph, 50 Stone Road East, Guelph, ON N1G 2W1, Canada; cpriyana@uoguelph.ca
  ^2^  Department of Agronomy and Plant Breeding, College of Agriculture and Natural Resources, University of Tehran, Karaj 31587-77871, Iran; mghorbani_90@ut.ac.ir
  ^3^  Genetics and Agricultural Biotechnology Institute of Tabarestan, Sari Agricultural Sciences and Natural Resources University, Sari 4818166996, Iran; e.bakhshandeh@sanru.ac.ir
  ^4^  Julius Kühn-Institut, Federal Research Centre for Cultivated Plants, Institute for Crop and Soil Sciences, Bundesallee 69, 38116 Braunschweig, Germany; z_rice_b@yahoo.com
  ^5^  International Faculty of Applied Technology, Yibin Unversity, Yibin 644000, China
  *  Correspondence: seyedh@uoguelph.ca (S.M.H.); mnaghavi@ut.ac.ir (M.R.N.)

Besides, we updated the Section “Author Contributions”:

**Before updating:**

Conceptualization, S.M.H.; validation, M.G. and S.M.H.; formal analysis, S.M.H. and P.Z.; data curation and visualization, M.G. and C.P.; writing—review and editing, S.M.H., C.P. and P.Z.; project administration, S.M.H. and M.R.N. All authors have read and agreed to the published version of the manuscript.

**After updating:**

Conceptualization, S.M.H.; validation, E.B., M.G. and S.M.H.; formal analysis, S.M.H. and P.Z.; data curation and visualization, M.G. and C.P.; writing—review and editing, S.M.H., E.B.; C.P. and P.Z.; project administration, S.M.H. and M.R.N. All authors have read and agreed to the published version of the manuscript.

The authors apologize for any inconvenience caused and state that the change does not affect the results of the study and the conclusions drawn from it. The original publication has also been updated.
